# Advancing chimeric antigen receptor T cell therapy with CRISPR/Cas9

**DOI:** 10.1007/s13238-017-0410-x

**Published:** 2017-04-22

**Authors:** Jiangtao Ren, Yangbing Zhao

**Affiliations:** Center for Cellular Immunotherapies, Perelman School of Medicine, University of Pennsylvania, Philadelphia, PA 19104-5156 USA

**Keywords:** CRISPR/Cas9, chimeric antigen receptor, T lymphocytes, adoptive immunotherapy, gene therapy

## Abstract

The clustered regularly interspaced short palindromic repeats (CRISPR)/CRISPR-associated 9 (CRISPR/Cas9) system, an RNA-guided DNA targeting technology, is triggering a revolution in the field of biology. CRISPR/Cas9 has demonstrated great potential for genetic manipulation. In this review, we discuss the current development of CRISPR/Cas9 technologies for therapeutic applications, especially chimeric antigen receptor (CAR) T cell-based adoptive immunotherapy. Different methods used to facilitate efficient CRISPR delivery and gene editing in T cells are compared. The potential of genetic manipulation using CRISPR/Cas9 system to generate universal CAR T cells and potent T cells that are resistant to exhaustion and inhibition is explored. We also address the safety concerns associated with the use of CRISPR/Cas9 gene editing and provide potential solutions and future directions of CRISPR application in the field of CAR T cell immunotherapy. As an integration-free gene insertion method, CRISPR/Cas9 holds great promise as an efficient gene knock-in platform. Given the tremendous progress that has been made in the past few years, we believe that the CRISPR/Cas9 technology holds immense promise for advancing immunotherapy.

## **INTRODUCTION**

The clustered regularly interspaced short palindromic repeats (CRISPR)/CRISPR-associated 9 (Cas9) system, a versatile RNA-guided DNA targeting technology, is triggering a revolution in the field of biology. CRISPR/Cas9 has demonstrated great potential for genetic manipulation, even in previously difficult contexts. Here, we review the current development of CRISPR/Cas9 technologies for therapeutic applications, especially chimeric antigen receptor (CAR) T cell-based adoptive immunotherapy. We compare the different methods used to facilitate efficient CRISPR delivery and gene editing in T cells. We explore the potential for genetic manipulation using the CRISPR/Cas9 system to generate universal CAR T cells and potent T cells resistant to exhaustion and inhibition. We discuss safety concerns regarding the specificity and future directions of CRISPR in the field of CAR T cell immunotherapy.

## **THE CRISPR/CAS9 SYSTEM**

The field of genome editing is evolving rapidly. Until a decade ago, zinc-finger nucleases (ZFNs) were the only practical option available for targeted genome editing (Bibikova et al., 2002, 2003; Porteus and Baltimore, 2003; Urnov et al., 2005; Morton et al., 2006; Doyon et al., 2008; Kim et al., 2009; Townsend et al., 2009). Zinc finger proteins recognize target DNA in a modular fashion: each protein consists of at least three zinc finger domains, and a single zinc finger domain interacts with a 3-bp sequence, making them ideal programmable sequence-specific DNA-binding proteins (Pavletich and Pabo, 1991). In 2011, transcription activator-like effector nucleases (TALENs) emerged as a competitive alternative to ZFNs (Boch et al., 2009; Moscou and Bogdanove, 2009; Cermak et al., 2011; Miller et al., 2011; Briggs et al., 2012). Unlike zinc fingers, each repeat domain in TALE proteins recognizes a single base. Four different repeat domains can be mixed and matched to create new DNA-binding proteins, which can be linked to the FokI domain to create a new class of programmable target DNA nucleases (Miller et al., 2011). These molecules enable precise targeting and cutting at a specific genomic locus to generate double-strand breaks (DSBs) followed by nonhomologous end joining (NHEJ) or homology-directed repair (HDR)-mediated repair, thereby enabling precise genome editing. Studies using these two classes of nucleases have led to important scientific discoveries and therapeutic development. In fact, a ZFN-based treatment of HIV that disables the HIV co-receptor C-C chemokine receptor type 5 (CCR5) in human primary T cells is currently in clinical trials and has shown great promise (Perez et al., 2008; Tebas et al., 2014). However, the recognition of the target DNA sequence by these protein-based genome engineering systems is determined by protein sequences. Tedious and complex protein engineering and optimization are therefore required for each specific target DNA sequence, and delivering many of these proteins into cells for simultaneous multiplexed genetic manipulation is challenging. Given these difficulties, their use for large-scale genomic manipulation or genetic screens has been limited.

The CRISPR/Cas9 technology originates from type II CRISPR/Cas9 systems, which provide bacteria with adaptive immunity to viruses, plasmids, and other foreign nucleic acids (Barrangou et al., 2007; Horvath and Barrangou, 2010; Wiedenheft et al., 2012). Type II CRISPR systems incorporate sequences from invading DNA between CRISPR repeat sequences that are encoded as arrays within the bacterial host genome. Transcripts from the CRISPR repeat arrays are processed into CRISPR RNAs (crRNAs) (Deltcheva et al., 2011), each containing a variable sequence transcribed from the invading DNA, which is known as the “protospacer” sequence, and part of the CRISPR repeat. Each crRNA hybridizes with a second RNA, which is known as the transactivating CRISPR RNA (tracrRNA) (Deltcheva et al., 2011), and these two RNAs form a complex with the Cas9 DNA endonuclease (Jinek et al., 2012). The protospacer-encoded portion of the crRNA guides Cas9 to complementary target DNA sequences and cleaves the DNA if they are adjacent to short sequences known as protospacer adjacent motifs (PAMs). The type II CRISPR system from Streptococcus pyogenes has been adapted for inducing sequence-specific DSBs and targeted genome editing. In 2012, Jinek et al. first demonstrated that the Cas9 protein from Streptococcus pyogenes (SpCas9) can bind with a tracrRNA-crRNA RNA complex to induce DSBs *in vitro* at a target DNA sequence by Watson-Crick base pairing of crRNA and target DNA (Jinek et al., 2012). This study also showed that directing Cas9 to bind and cleave a specific DNA sequence did not require an RNA complex. The process can be simply achieved by using a designed, chimeric single guide RNA (sgRNA). In 2013, two groups from MIT and Harvard demonstrated the feasibility of genome editing of human cells using the CRISPR/Cas9 system (Cong et al., 2013; Mali et al., 2013b). These discoveries paved the way and opened the era for the use of CRISPR/Cas9 in genome engineering, including gene editing and gene expression regulation, epigenetic modification, and genome imaging (Cheng et al., 2013; DiCarlo et al., 2013; Gilbert et al., 2013; Hwang et al., 2013; Li et al., 2013; Maeder et al., 2013; Nekrasov et al., 2013; Perez-Pinera et al., 2013; Qi et al., 2013; Shen et al., 2013; Wang et al., 2013; Tanenbaum et al., 2014; Chavez et al., 2015; Hilton et al., 2015; Kearns et al., 2015; Konermann et al., 2015).

## **GENE EDITING AND THERAPEUTIC APPLICATION OF CRISPR/CAS9 IN HUMAN T CELLS**

In addition to generating powerful research tools, genome editing with CRISPR/Cas9 technology holds great promise as a means to produce therapeutic agents or as a therapeutic itself. Although we focus on SpCas9, particularly its use in therapeutic applications and the development of next-generation transformational drugs in T cells, the general outline described here applies to the larger ensemble of CRISPR/Cas9 tools.

The therapeutic potential of CRISPR/Cas9 has already been demonstrated in many aspects. CRISPR/Cas9 has been applied as an antimicrobial agent and has been developed to specifically target antibiotic resistance in highly virulent strains of bacteria (Makarova et al., 2006). Gene therapy applications have also been tested for monogenic diseases. A CFTR gene defect was repaired in cells from human patients with cystic fibrosis *in vitro* in cultured intestinal stem cell organoids using CRISPR-Cas (Schwank et al., 2013). Correction of the defective gene causing hereditary tyrosinaemia was performed in mice after the hydrodynamic injection of CRISPR components. This application led to an expansion of mutation-corrected hepatocytes *in vivo* and resulted in a rescued phenotype in adult mice (Yin et al., 2014). Advancing from the described therapeutic treatment to preventative techniques, muscular dystrophy was prevented via germ line gene editing (Long et al., 2014). The use of CRISPR/Cas9 to treat viral infections, such as HIV and hepatitis B, has also been demonstrated (Zhen et al., 2015). IPSC resistant to HIV-1 was also generated through genome editing (Hu et al., 2014; Ye et al., 2014).

The application of genome editing for therapeutic purpose has begun to overlap with the rapidly evolving field of cancer immunotherapy, particularly for the production of next-generation chimeric antigen receptor (CAR) T cells. These modified T cells armed with tumour-targeting receptors have demonstrated great promise in clinical trials treating various leukaemias and lymphomas and may eventually be used to treat solid cancers (Maus et al., 2014). CARs comprise an extracellular single-chain variable fragment (ScFv) specific to an antigen on tumour cells and an intracellular chimeric signalling domain that drives T cell activation and the killing of tumour cells (Gross et al., 1989; Irving and Weiss, 1991; Maher et al., 2002; Brentjens et al., 2003; Carpenito et al., 2009). To date, the best CAR T cell therapy involves targeting CD19, an antigen expressed by B cells and B cell malignancies. Several other CAR T therapies targeting solid tumours antigens, such as Her2/neu, Mesothelin cMet, GD2, interleukin-13 receptor alpha 2 (IL13Rα2), CEA, and EGFR, are currently under evaluation in different phases of clinical trials.

Currently, most CAR T clinical trials utilize autologous T cells and might therefore be hampered by the poor quality and quantity of T cells and by the time and expense of manufacturing autologous T cell products. CAR T cell therapy could substantially benefit from allogeneic universal donor T cells, as “off-the-shelf” cells could greatly increase the number of patients who could be treated by a single CAR T cell product. However, endogenous TCR on allogeneic T cells may recognize the alloantigens of the recipient, leading to graft-versus-host disease (GVHD); furthermore, the expression of HLA on the surface of allogeneic T cells causes rapid rejection by the host immune system. In this context, ZFNs and TALENs have been used to knock out endogenous T cell receptor genes in T cells, which could prevent unwanted graft-versus-host reactivity (Provasi et al., 2012; Torikai et al., 2012; Poirot et al., 2015). Genome-editing strategies could also be used to prevent or delay the rejection of CAR T cells by the recipient’s immune system by eliminating or decreasing the expression of histocompatibility antigens on the donor T cells. Future CAR T cell therapies could benefit from combined modification of endogenous TCR genes, histocompatibility genes, and components of signalling pathways. In a previous study, we reported the use of the CRISPR/Cas9 system to simultaneously disrupt multiple genomic loci. CAR T cells deficient in the expression of endogenous T cell receptor (TCR) and HLA class I (HLA-I) were generated that can be used as universal CAR T cells (Ren et al., 2016).

In addition to enabling the generation of universal CAR T cells, genome editing could be used to enhance CAR T cell function by ablating the genes encoding T cell inhibitory receptors or signalling molecules, such as programmed cell death protein 1 (PD1) or cytotoxic T lymphocyte-associated protein 4 (CTLA4) (Lloyd et al., 2013; Hoos, 2016; Su et al., 2016). Indeed, a clinical trial has recently been approved by the US National Institutes of Health (NIH) Recombinant DNA Advisory Committee (RAC) that will be conducted at the University of Pennsylvania. In this clinical trial, PD1 and the endogenous TCR will be knocked out by CRISPR/Cas9 in NY-ESO-1 TCR transduced T cells. The first clinical trial of CRISPR/Cas9 has been initiated. The trial uses CRISPR/Cas9 to knock out PD1 in T cells of patients with lung cancer; however, CAR or TCR will not be introduced into T cells in this trial (Cyranoski, 2016). Similar trials with PD1-knockout autologous T cells for prostate (NCT02867345), bladder cancer (NCT02863913), and renal cell carcinoma (NCT02867332) are also being initiated. Scientists are seeking to introduce CAR via HDR to eliminate the need to randomly integrate viral delivery systems and control where CAR integrates (Sadelain et al., 2011; Kalos and June, 2013). Notably, simply ablating inhibitory molecules can be a double-edged sword. It is important to investigate whether the removal of some inhibitory signals from the T cells leads to the uncontrolled proliferation of cells or to severe autoimmunity.

## **DELIVERY OF CRISPR/CAS9 IN T CELLS**

The CRISPR/Cas9 system can be directly applied to human cells by transfection with a plasmid that encodes Cas9 and sgRNA (Cong et al., 2013). The viral delivery of CRISPR components has been extensively demonstrated using lentivirus and retrovirus (Shalem et al., 2014; Williams et al., 2016). Gene editing with CRISPR encoded by non-integrating virus, such as adenovirus and adenovirus-associated virus (AAV), has also been reported (Ran et al., 2015; Swiech et al., 2015). Recent discoveries of smaller Cas proteins have enabled and enhanced the combination of this technology with vectors that have gained increasing success for their safety profile and efficiency, such as AAV vectors. Due to their relatively low immunogenicity, AAVs are commonly chosen for *in vivo* gene delivery in somatic gene therapy (Friedland et al., 2015; Ran et al., 2015). CRISPR delivery via Cas9 ribonucleoproteins (RNP) also exhibited efficient gene editing in human cells (Kim et al., 2014).

Gene disruption in T cells had been achieved by lentiviral and adenoviral delivery of CRISPR components into primary T cells. However, these methods cannot site-specifically insert and occasionally disrupt essential genetic elements, and the gene disruption efficiency was not very high (Wang et al., 2014; Li et al., 2015). Recently, a Jurkat T cell-based lentiviral CRISPR toolbox was developed to facilitate the research on T cell function. Given the flexible and easy-to-handle features of Jurkat T cells, and programmability with different Cas9 variants, the toolbox might serve as a useful platform for the study of T cell signal transductions (Chi et al., 2016). Although gene ablation in T cells with DNA nucleofection of CRISPR reagents was also achieved, DNA nucleofection is associated with high toxicity to T cells, which represents a major difficulty for its application (Mandal et al., 2014; Su et al., 2016). Schumann et al. reported the site-specific genome editing of primary human T cells using Cas9 RNPs (Schumann et al., 2015). Cas9 RNPs are recombinant Cas9 proteins complexed with *in vitro*-transcribed sgRNAs. Cas9 RNPs delivered via electroporation efficiently ablate CXCR4 and PD-1 expression in CD4 T cells by introducing insertions or deletions (indels) in the targeted region. Furthermore, the inclusion of a HDR template successfully introduced exogenous DNA into the genome at the Cas9 cleavage site. Deep sequencing results indicated that up to 55% of the treated cells contained indels in the targeted region, with 20% of the cells incorporating the exogenous DNA sequence introduced through the HDR template.

The work by Schumann et al. is joined by other recent reports focused on primary human T cell gene editing using CRISPR/Cas9. Hendel et al. reported the disruption of CCR5 locus in T cells by co-delivering Cas9 mRNA or protein with chemically modified sgRNAs via electroporation, achieving up to 49% target mutagenesis in activated primary human T cells calculated by tracking of indels by decomposition (TIDE) analysis (Hendel et al., 2015).

**Figure 1:**
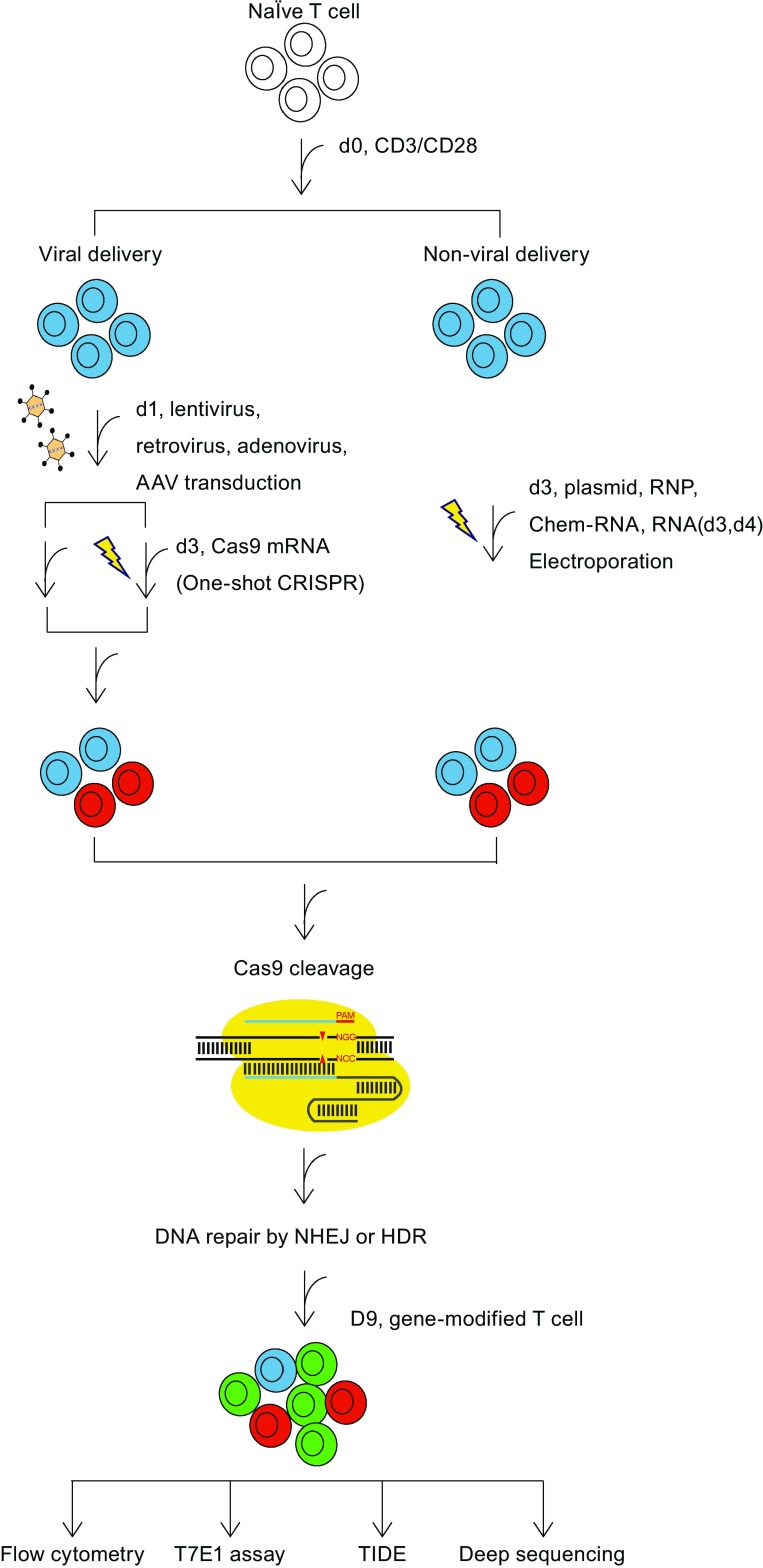
**A brief summary of various delivery methods of CRISPR/Cas9 components into T cells**

A major challenge in primary T-cell engineering lies in the limited time frame in which genetic manipulation can be accomplished with high efficiency. Unstimulated primary or naïve T cells are significantly less receptive to exogenous nucleic acid or protein uptake compared with stimulated T cells (Hendel et al., 2015). By contrast, repeated stimulation will lead to T-cell exhaustion and decrease its anti-tumour efficacy. Therefore, protocol optimization will be required to enable the effective application of multiple genetic manipulation techniques on one T-cell product. To this end, we recently reported a method to incorporate disrupting endogenous genes into standard clinical CAR T cell manufacturing processes (Ren et al., 2016). Freshly isolated human T cells were stimulated via anti- CD3/CD28 beads and lentivirally transduced at 1 day post-stimulation to stably express a CAR transgene, and T cells were electroporated at days 3 and 4 with RNA-encoding Cas9 and sgRNA to disrupt TCR, HLA-I, and PD1 simultaneously. The editing efficiency using this combined protocol was donor dependent, with the results indicating >70% CAR transduction efficiency and >60% double-knockout efficiency in most production runs. This manufacturing procedure yielded CAR T cells that were specific to CD19 targets, resistant to host rejection, and incapable of triggering GVHD, thus highlighting the ability to generate multi-functional universal CAR T cells with CRISPR/Cas9 techniques. Similar results were also reported using another approach of CRISPR/Cas9 RNPs targeting the same 3 genes: TCR, B2m, and PD1 (Liu et al., 2017). Compared to multiple deliveries of sgRNAs, multiplex genome editing with Cas9 RNP in T cells reduced the toxicity associated with RNA electroporations at the cost of decreased gene targeting efficiency. The authors used multiple sgRNAs targeting the same gene to improve gene disruption efficiency, which may potentially increase the off-target effects. To further improve the application of multiplex genome editing and to reduce the toxicity associated with multiple electroporation, we developed a One-shot CRISPR system, by incorporation of multiple gRNAs in a CAR lentiviral vector. Efficient multiple gene modification can be achieved by a single electroporation of various Cas9 mRNAs (Ren et al., 2017). A brief summary of various delivery methods of CRISPR/Cas9 components into T cells is schematically presented in Fig. 1. A comparison between different methods for CRISPR gene editing in T cell is presented in Table 1.

**Table 1:** Comparison of various delivery methods of CRISPR/Cas for T cell gene editing

	Viral-delivery				Non-viral-delivery			
	Integrating virus		Integrating-free virus		Single electroporation		Double electroporation	
	Lentivirus	Retrovirus	Adenovirus	AAV	Plasmid	RNP	Chem-RNA	RNA
Integration	Yes	Yes	No	No	Rear	No	No	No
Efficiency	Low	Low	Low	Low	Low	Medium	Medium	High
Electroporation	No	No	No	No	Yes	Yes	Yes	Yes
Toxicity	Medium	Medium	Low	Low	High	Low	Low	Medium
Off-target effects	Medium	Medium	Low	Low	Medium	Rear	Low	Rear

RNP: Cas9 protein-sgRNA complex; Chem-RNA: Cas9 protein or mRNA complexed with chemically modified sgRNA; RNA: Cas9 mRNA and sgRNA

These Cas9-based gene-editing techniques will enable the disruption of a wide variety of target genes, including endogenous TCR, the checkpoint receptors PD1 or CTLA-4 in tumour-targeting T cells or the virally targeted chemokine receptors CCR5 and CXCR4 in T cells of HIV patients. The insertion of exogenous DNA sequences using HDR templates demonstrated by Schumann et al. further opens the possibility of precisely integrating transgenic elements, thereby reducing the risk of gene integration in oncogenic sites caused by viruses. However, the current efficiency of site-specific gene knock-in by nuclease-mediated homologous recombination is not comparable to that of standard viral transduction. Thus, the next step for gene editing in T cells will be to develop protocols that enable the combinatorial application of various gene-editing techniques in primary T cells.

## **SAFETY ISSUES AND CONCERNS**

The off-target activities of Cas9 can be measured by directly assessing the potential off-target genomic DNA sites defined by the sequences that have 1–6 nucleotide (nt) differences to the intended target sequence. A given 20-nt target sequence might have hundreds of such potential off-targets within the human genome. The T7 Endonuclease I (T7E1) mutation mismatch assay is commonly used to detect high indel frequencies (>2%–5%). As a result, more sensitive deep sequencing assays are needed to identify lower frequency off-target mutations (Cho et al., 2014; Fu et al., 2014). Exome sequencing was also used for off-target analysis, but the high false negative result rate associated with exome sequencing analysis limits its interpretation of the results. However, accurately predicting the off-target cleavage sites remains a challenge because it is typically biased, given the inability of most available algorithms for off-target prediction to cover all potential off-target sites. The genome-wide detection of DSBs provides a non-biased method to assess the specificity of Cas9-mediated DNA cleavage, and several methods have been developed to meet this purpose. In one method called genome-wide unbiased identification of DSBs enabled by sequencing (GUIDE-Seq), the Cas9-sgRNA induced DSBs are tagged in the genomes of living cells by introducing a blunt, double-stranded oligodeoxynucleotide during the end-joining process following a DSB. The double-stranded oligodeoxynucleotide integration sites are then amplified and deep sequenced (Tsai et al., 2015). A modified high-throughput, genome-wide translocation sequencing (HTGTS) was developed based on linear-amplification-mediated PCR (LAM-PCR HTGTS). LAM-PCR HTGTS enables the detection of DSBs based on translocation to other endogenous or ectopic DSBs using the target DSB as “bait” to capture the “prey” sequences translocated to the target DSB (Frock et al., 2015). A third method, called *in situ* breaks labelling, enrichments on streptavidin, and next generation sequencing (BLESS), captures biotinylated oligonucleotides labelled DSBs in fixed cells using streptavidin. Enriched DSB-containing DNA fragments are PCR amplified and analysed by deep sequencing (Crosetto et al., 2013; Ran et al., 2015). A fourth method called digested genome sequencing (Digenome-Seq) uses isolated genomic DNA for *in vitro* Cas9-mediated digestion followed by whole-genome sequencing to evaluate genome-wide Cas9 off-target effects (Kim et al., 2015).

CRISPR/Cas9 generally disrupts their intended target sites reliably; however, an important question to consider is to what extent these nucleases induce off-target cleavage events, especially in therapeutic application. CRISPR/Cas9 gene editing generates off-target mutations depending upon the experimental setting and cell type (Cho et al., 2014). Pankaj et al. reported an extremely low incidence of off-target mutagenesis of CRISPR in hematopoietic stem cells (Mandal et al., 2014). Recent studies also demonstrated a low incidence of off-target mutagenesis in T cells using lentivirus and adenovirus-delivered CRISPR/Cas9 to knockout CCR5 (Wang et al., 2014; Li et al., 2015). Another report showed no detectable off-target mutations in the CXCR4-knockout CD4 T cells (Hou et al., 2015). We reported rare off-target mutagenesis targeting TRAC or TRBC with Cas9 (Ren et al., 2016). Although these studies all suggest that T cells might be minimally tolerable to CRISPR/Cas9-induced off-target mutagenesis, non-biased strategies combined with deep sequencing for off-target detections should be applied to the selected target gRNA used in the clinical trials.

## **FUTURE DIRECTIONS**

Reducing the off-target effects for safe therapeutic application of CRISPR/Cas9 in immunotherapy remains unresolved. Various approaches have been explored to improve the specificity of CRISPR/Cas9. The choice of proper target sequence is the first and most effective option to improve the specificity. All published studies have suggested that the CRISPR/Cas9 mediated off-target mutagenesis could vary depending on the sgRNA design and target sequence. Predictive algorithms have been developed to facilitate this process by computationally searching target sequences that bear the least similarities to other sequences to reduce the off-target effects.

Precisely tuning the amount of Cas9 and sgRNA in cells is also used to improve the specificity; some studies have demonstrated that a decrease in the amount of CRISPR reagents in cells could reduce off-target effects (Fu et al., 2013; Hsu et al., 2013). Timely controlled Cas9 expression is also demonstrated through a tet-on system (Gonzalez et al., 2014). Furthermore, gene editing with the Cas9 mRNA and protein causes fewer off-target effects compared to plasmids and viruses, likely because the mRNA or RNPs were rapidly degraded after immediate on-target cleavage (Kim et al., 2014). Modifying the sgRNA sequence also improved the specificity. For example, sgRNA with a truncated base-pairing sequence (17 nt instead of 20 nt) enhanced the targeting specificity because truncated sgRNAs have reduced binding affinity with the target DNA and thus are more sensitive to mismatches (Fu et al., 2014).

An alternative approach is to take advantage of the Cas9 nickase that contains mutations in one of the two nuclease domains, HNH or RuvC, which cleave the DNA strand complementary and noncomplementary (respectively) to the sgRNA (Gasiunas et al., 2012; Jinek et al., 2012). A pair of Cas9 nickases could generate two single-strand breaks adjacent to each other on opposite DNA strands when guided by two properly designed sgRNAs (Mali et al., 2013a; Ran et al., 2013; Cho et al., 2014). The paired nickases exhibit higher specificity in editing because the generation of DSBs requires two independent binding events, whereas the nuclease Cas9 requires only one binding event. A similar strategy is to fuse DNA-endonuclease-dead Cas9 (dCas9) to the dimerizing FokI nuclease. The dCas9-FokI fusion is an RNA-guided nuclease that cleaves DNA only when a pair of FokI domains is sufficiently close to form a dimer. Efficient cleavage occurs when two target sites are spaced approximately 13–25 bp apart (Guilinger et al., 2014; Wyvekens et al., 2015). Moreover, because the FokI nuclease activity relies on dimerization, this strategy also reduced unwanted mutagenesis compared to the Cas9 nickase (Guilinger et al., 2014; Wyvekens et al., 2015). However, these approaches improve CRISPR specificity at the cost of reduced efficiency.

Facilitated by the crystal structure of SpCas9, two recent studies have reported more precise genomic edits with rationally engineered CRISPR/Cas9 systems. Slaymaker et al. created systematic single or multiple mutations in the positively charged residues that are predicted to be involved in the interaction with the non-target strand of the target DNA and identified Cas9 mutants that decrease off-target effects without impairing on-target activity (Slaymaker et al., 2016). Using a similar approach, Kleinstiver et al. made a quadruple amino acid substituted SpCas9 that retains high on-target activity with minimal off-target activity. Application and further exploration of high-fidelity Cas9 variants will increase the reliability of CRISPR/Cas9 as both a research tool and a therapeutic approach.

As an integration-free gene insertion method, CRISPR/Cas9 holds great promise as an efficient gene knock-in platform. Conventional CAR expression in T cells requires randomly integrating viral delivery vectors, including lentivirus and retrovirus. However, uncontrolled virus integration in host cell genomes has the potential risk of causing insertional mutagenesis. CRISPR/Cas9 mediates efficient gene knock-in in human cells, embryos, and plants. Schumann et al. reported successful introduction of exogenous DNA into T cells genome at the Cas9 cleavage site. Although the knock-in of large fragments, such as a CAR in T cells, remains challenging, doing so is desirable for therapeutic application and needs further exploration. Sather et al. reported the feasibility of knock-in of a CAR transgene in T cells with MegaTal and an AAV HDR template (Sather et al., 2015). A more recent study showed that targeting a CAR to the TRAC locus greatly enhanced the antitumor activity by reducing tonic activation (Eyquem et al., 2017). Microhomology Mediated End Joining (MMEJ) has been used for gene knock-in in human cells and animals wherein large homology arm is not required, thus facilitating gene delivery with small inserts (Nakade et al., 2014; Sakuma et al., 2015). Previously, some studies have highlighted the possibility of knock-in of large gene cassettes using homology-independent targeted integration strategy, which enables robust DNA knock-in in both dividing and non-dividing cells *in vitro* and, more importantly, *in vivo* (Auer et al., 2014; He et al., 2016; Suzuki et al., 2016).

Given the tremendous progress that has been made in the past several years, we believe that the CRISPR/Cas9 technology holds immense promise for advancing immunotherapy.
